# Idiopathic Transverse Myelitis Mimicking an Intramedullary Spinal Cord Tumor

**DOI:** 10.1155/2016/8706062

**Published:** 2016-09-08

**Authors:** Andrew A. Fanous, Nathan P. Olszewski, Lindsay J. Lipinski, Jingxin Qiu, Andrew J. Fabiano

**Affiliations:** ^1^Department of Neurosurgery, Jacobs School of Medicine and Biomedical Sciences, University at Buffalo, State University of New York, Buffalo, NY, USA; ^2^Jacobs School of Medicine and Biomedical Sciences, University at Buffalo, State University of New York, Buffalo, NY, USA; ^3^Department of Pathology, Roswell Park Cancer Institute, Buffalo, NY, USA; ^4^Department of Neurosurgery, Roswell Park Cancer Institute, Buffalo, NY, USA

## Abstract

The differential diagnoses for spinal cord lesions include spinal tumors and inflammatory processes. The distinction between these pathologies can be difficult if solely based on imaging. We report for the first time to our knowledge a case of idiopathic transverse myelitis (ITM) mimicking a discrete cervical spinal lesion in a 66-year-old man who presented with gait instability and neck pain. The patient's symptoms failed to resolve after an initial course of steroid therapy. Surgical biopsy confirmed the diagnosis of ITM. Subsequent treatment with dexamethasone resulted in complete resolution of the symptoms as well as the intramedullary enhancement. ITM is most common in the cervical and thoracic spine, spanning 3-4 spinal segments. It usually occupies more than 50% of the cross-sectional area of the spinal cord and tends to be central, uniform, and symmetric. It exhibits patchy and peripheral contrast enhancement. These criteria are useful guidelines that help distinguish ITM from neoplastic spinal lesions. A decision to perform biopsy must take into consideration the patient's clinical symptoms, the rate of progression of neurological deficits, and the imaging characteristics of the lesion. Surgical biopsy for questionable lesions should be reserved for patients with progressive neurological deficits refractory to empirical medical therapy.

## 1. Introduction

The differential diagnosis for spinal cord mass lesions includes spinal cord tumors, inflammatory disease processes, and infectious entities. Making the distinction among these pathologic entities can be difficult solely on the basis of imaging modalities and clinical symptoms. As a result, surgical biopsy procedures are often performed on questionable intramedullary spinal cord mass lesions [1].

Transverse myelitis is an umbrella term that encompasses multiple infectious and inflammatory disease processes that present with symptoms and imaging findings consistent with spinal cord compression [2]. When all other diseases are excluded through diagnostic and laboratory investigations, the diagnosis of idiopathic transverse myelitis (ITM) is made. In this paper, we report the case of a patient who presented with idiopathic transverse myelitis mimicking a spinal cord tumor on neuroimaging. To the best of our knowledge, this is the first time a case of ITM mimicking a discrete spinal lesion has been reported.

This case report illustrates the diagnostic dilemma that faces radiologists, neurologists, and neurosurgeons who care for patients afflicted by rapid neurological deterioration and imaging findings consistent with intramedullary spinal cord mass lesions. This report also provides a review of the current literature discussing inflammatory disease processes that mimic spinal cord tumors. In addition, it discusses the most important features that help distinguish the two groups of disease on magnetic resonance imaging (MRI).

## 2. Case Presentation

**Figure 1:**
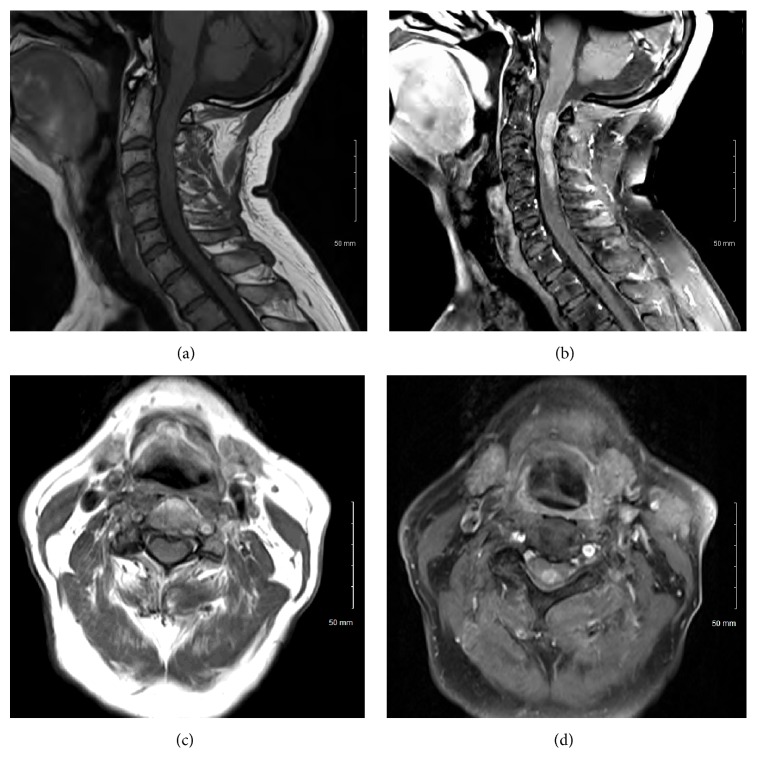
Preoperative T1-sequence MRI of the cervical spine. Sagittal images without (a) and with (b) gadolinium contrast material and axial images without (c) and with (d) gadolinium, showing an eccentric heterogeneously gadolinium-enhancing intramedullary mass between C2 and C4.

**Figure 2:**
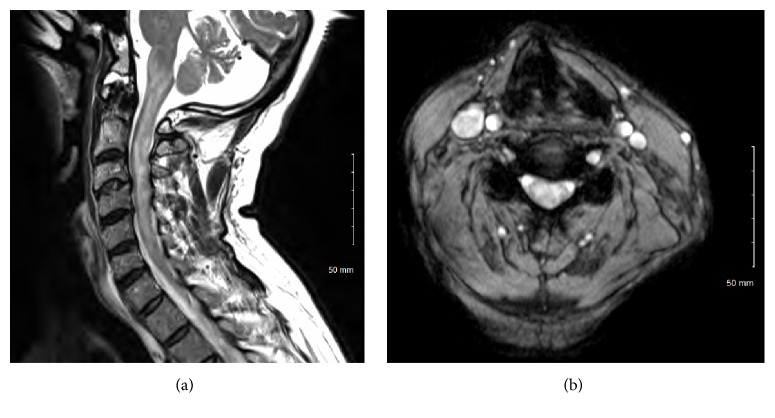
Preoperative T2-sequence MRI of the cervical spine. Sagittal (a) and axial (b) images showing diffuse spinal cord edema and T2-signal changes around the lesion.

A 66-year-old man presented to our clinic with a 4-week history of balance difficulty, as well as pain in the neck and both shoulders. Physical examination revealed diffuse hyperreflexia but normal strength in all 4 extremities. Sensation was intact in the arms but decreased to light touch, vibration, and proprioception in the legs bilaterally. Sensation to pinprick was unremarkable. The patient's gait was significantly ataxic. Visual examination was unremarkable. MRI of the cervical spine revealed the presence of an eccentric heterogeneously gadolinium-enhancing intramedullary mass between C2 and C4 (Figure 1). Diffuse surrounding spinal cord edema was also evident (Figure 2). MRI of the thoracic and lumbar spine as well as of the brain did not reveal any evidence of other lesions. A lumbar puncture was performed, and analysis of the cerebrospinal fluid (CSF) revealed mild pleocytosis (red blood cell count 48 cells/*μ*L, leukocyte count 13 cells/*μ*L with 93% lymphocytes), normal glucose (54 mg/dL), and normal protein (93 mg/dL). The CSF was negative for oligoclonal bands and myelin basic protein. The patient's symptoms improved with oral dexamethasone therapy (6 mg taken every 6 hours) but did not completely resolve. Repeat cervical spine MRI 3 weeks after the initiation of dexamethasone therapy demonstrated no change in lesion size or enhancement pattern. The case was presented at a multidisciplinary tumor board meeting, and biopsy and possible resection were recommended. After discussion of the various options and their associated risks and benefits, the patient and his family opted for surgical intervention.

**Figure 3:**
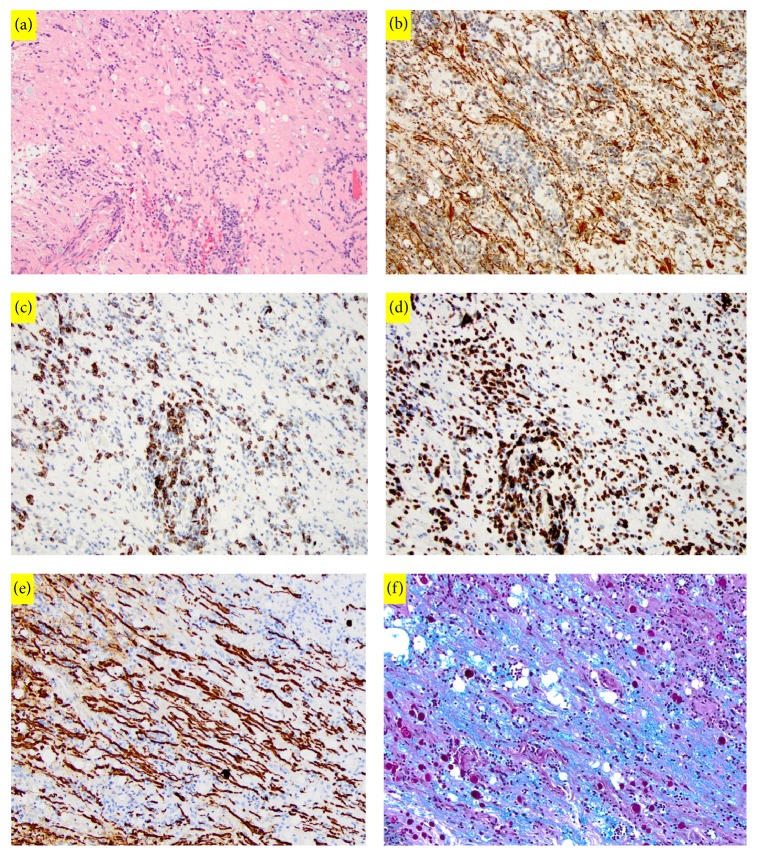
(a) Representative H&E section of lesional tissue (200x). There are perivascular and parenchymal infiltrates of lymphocytes. The blood vessel wall shows hyalinization changes and there is congestion in the capillaries. (b) Glial fibrillary acidic protein (GFAP) immunostains show focal gliosis (200x). (c) A few CD20 positive B cells are present (200x). (d) A few CD3 positive T cells are present (200x). (e) Neurofilament immunostain highlights axons (200x). (f) Luxol fast blue- (LFB-) periodic acid-Schiff (PAS) stains show intact myelin (200x). There is no evidence of glioma, lymphoma, demyelinating disease, granuloma, viral-cytopathic changes, fungal organisms, or mycobacterial organisms.

**Figure 4:**
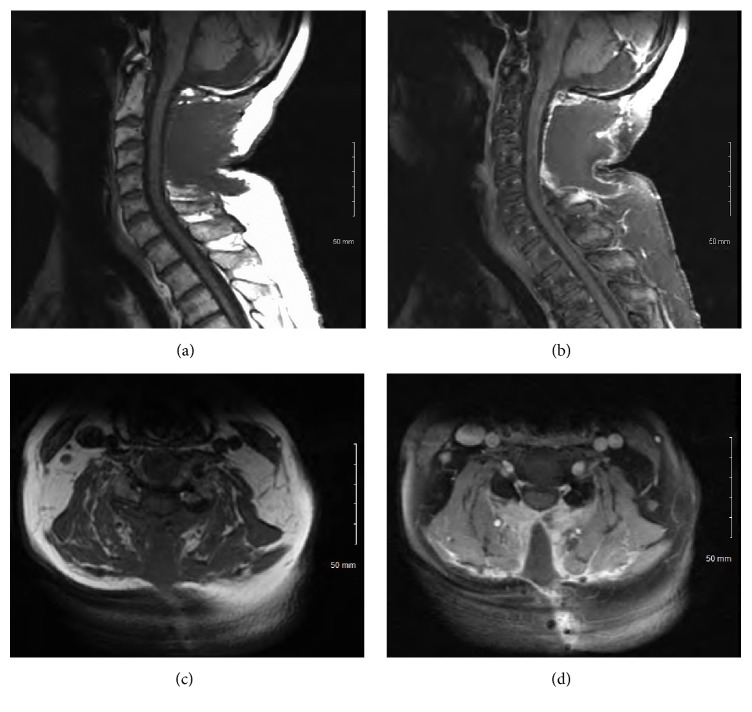
T1-sequence MRI of the cervical spine obtained 2 months postoperatively. Sagittal images without (a) and with (b) gadolinium contrast material and axial images without (c) and with (d) gadolinium, showing complete resolution of the enhancing lesion.

**Figure 5:**
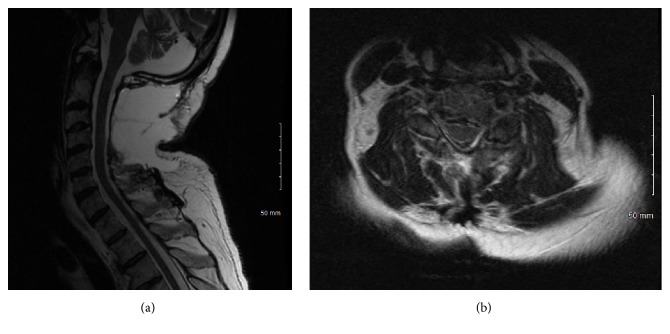
Postoperative T2-sequence MRI of the cervical spine. Sagittal (a) and axial (b) images, showing complete resolution of the preoperative intramedullary lesion and its surrounding T2-signal changes.

The patient subsequently underwent a C1 to C5 laminectomy with the use of intraoperative neuromonitoring. After opening the dura and arachnoid membranes, a bulging, discolored portion of the spinal cord was clearly identifiable near the midline. The lesion measured approximately 1.5 cm × 0.5 cm. A midline myelotomy was performed at this location, and abnormal tissue was sent for pathologic analysis. However, no clear plane of dissection could be identified. Pathologic analysis of the frozen specimen suggested the presence of inflammatory cells or possibly lymphoma. In light of these findings, a decision was made not to proceed with any further resection. The patient tolerated the procedure well and experienced improvement in his gait following the surgery. He was discharged home on the fifth postoperative day. The final pathology report was consistent with transverse myelitis; and there was no evidence of glioma, lymphoma, demyelinating disease, granuloma, viral-cytopathic changes, fungal organisms, or mycobacterial organisms (Figure 3). Luxol fast blue (LFB) staining of the sample showed intact myelin (Figure 3(f)). Further diagnostic tests revealed normal serum levels of neuromyelitis optica IgG autoantibodies, methylmalonic acid, vitamin B12, folate, angiotensin-converting enzyme, thyroid-stimulating hormone, anticardiolipin antibodies (IgG, IgM, and IgA), antiribonucleoprotein antibodies, and Smith antibodies. Serum tests were also negative for antinuclear antibodies and nonreactive for human immunodeficiency virus and rapid plasma reagin. The patient was treated with oral dexamethasone therapy, and MRI of the cervical spine obtained 2 months postoperatively showed complete resolution of the enhancing lesion (Figure 4) and its surrounding edema (Figure 5). His neurological examination returned to baseline and he no longer had any neurological deficits.

## 3. Discussion

The wide variety of spinal cord pathologies, clinical symptomatologies, patient demographics, and neuroimaging characteristics hinder preoperative attempts to distinguish neoplastic from inflammatory processes that present as spinal cord masses. Most intramedullary lesions in adults are neoplastic in nature [6]. Although nonneoplastic inflammatory processes of the spinal cord are uncommon, they make up a significant percentage of intramedullary spinal cord lesions that ultimately undergo surgical biopsy. For instance, in a large study conducted by Cohen-Gadol et al. [1] of 38 patients with questionable spinal lesions who underwent surgical biopsy, 53% of the lesions were consistent with inflammatory processes, whereas only 21% were neoplasms. In that study, the inflammatory processes included demyelinating diseases (21%), sarcoidosis (13%), chronic nonspecific inflammation (5%), eosinophilic vasculitis (3%), noncaseating granulomatous angiitis (3%), nonspecific histiocytic reaction (3%), schistosomiasis (3%), and tuberculosis (3%).

The central nervous system is the preferred target for several inflammatory conditions. Such conditions include viral and postviral infections (including human immunodeficiency virus and acute disseminated encephalomyelitis [ADEM]), systemic lupus erythematosus, Behçet's disease, Sjögren's syndrome, Wegener's granulomatosis, sarcoidosis, and neuromyelitis optica. “Transverse myelitis” is an umbrella term that encompasses all these conditions [2, 7]. When all other causes of transverse myelitis are excluded through laboratory investigation, the diagnosis of idiopathic transverse myelitis (ITM) is made [2, 7].

The presentation of transverse myelitis as a mass lesion that mimics a spinal cord tumor has been described in a paucity of reports in the neurological and neurosurgical literature. Cases found in the literature are summarized in Table 1. These 7 cases of inflammatory processes posing as a spinal mass share some common features [3–5]. All were found in middle-aged patients, with a mean age of 37 years. Five were women; only 2 were men. All lesions but one involved the cervical spine, and these lesions were most common in the upper cervical segments between C1 and C4. Clinically, patients most commonly presented with paresis, paresthesia, and urinary tract-related symptoms. The presence of oligoclonal bands was rather inconsistent (in 4 of 7 cases) and did not correlate with the final diagnosis. Four of the 7 patients underwent surgical biopsy procedures that confirmed the lack of any neoplastic process. The final diagnoses included ADEM (3 cases), neuromyelitis optica (NMO) (2 cases), and multiple sclerosis (MS) (2 cases).

In 2004, Dhiwakar and Buxton [3] reported the case of a 36-year-old woman who presented with a 10-day course of rapid neurological deterioration, culminating in quadriparesis, paresthesia, and urinary retention. Imaging revealed an intramedullary lesion extending from C1 to T2, with significant spinal cord enlargement, cystic changes, and minimal contrast enhancement. The CSF analysis at that time was unremarkable. Surgical exploration of the lesion revealed an indistinct, poorly demarcated lesion. Pathological examination revealed inflammatory changes with no evidence of malignancy or infection. A second CSF analysis revealed the presence of oligoclonal bands. Therefore, this was most likely a case of MS, in spite of the fact that the authors did not discuss it as such and only reported it as a case of transverse myelitis. However, oligoclonal bands in the CSF are typically absent in non-MS-related transverse myelitis [8, 9].

The exact etiology of ITM remains unknown, although it is postulated to be viral in origin, because many afflicted patients report flu-like symptoms prior to the onset of myelopathy [2, 10]. The incidence of ITM is approximately 1.5 to 4.5 per 1 million persons per year [11]. ITM is most common in the cervical and thoracic spine, spanning 3 to 4 spinal segments [2]. Furthermore, ITM tends to occupy more than 50% of the cross-sectional area of the spinal cord on MRI and tends to be central, uniform, and symmetric [10, 12]. It exhibits patchy and peripheral enhancement following the administration of gadolinium [12, 13].

Intramedullary tumors of the spinal cord can be very difficult to distinguish from other nonneoplastic lesions. This is demonstrated by the large series of 38 patients studied by Cohen-Gadol et al. [1], wherein only 47% of the preoperative diagnoses made by surgeons and/or neuroradiologists were correct and less than 50% of the lesions thought to be intramedullary tumors on imaging were found to be neoplastic on histopathological analysis. Conversely, mistaking an intramedullary neoplasm for an inflammatory process has been also reported in the literature, which further highlights the difficulty of establishing a diagnosis solely on the basis of imaging findings [7, 14].

The most common intramedullary spinal cord tumors are ependymomas and astrocytomas. These tumors appear as hyperintense lesions on T2 MRI sequences with associated focal enlargement of the spinal cord [15, 16]. In MS, MRI often exhibits small well-circumscribed intramedullary lesions that are hyperintense on T2 sequences (relapsing-remitting type of MS) or demyelination and atrophy (primary and secondary progressive MS) [17, 18]. Multiple lesions are often seen in MS. By contrast, ADEM exhibits long, centrally located demyelinating lesions that expand the length of two or more spinal cord segments [19, 20]. Spinal cord tumors may occur anywhere in the spinal cord, with ependymomas having a predilection for the conus medullaris and filum terminale and astrocytomas most commonly located in the upper thoracic spine [15, 16]. Conversely, inflammatory spinal cord diseases such as MS and ADEM are most commonly located in the cervical spine, as demonstrated in Table 1 [4, 17, 21]. Enlargement of the spinal cord on MRI is controversial, with some authors suggesting that lack of spinal cord enlargement correlates with nonneoplastic inflammatory processes and others reporting medullary expansion with such diseases [1, 4, 17]. Contrast enhancement is a nonspecific finding on MRI and does not correlate with the type of pathology that is present [1].

Furthermore, the clinical symptoms of intramedullary spinal tumors are difficult to distinguish from those of inflammatory process afflicting the spinal cord. These symptoms include weakness, paresthesia, bladder dysfunction, and occasionally pain. Although neurological symptoms associated with spinal tumors tend to be more indolent in course and progressive in nature, those associated with inflammatory processes tend to be more acute [7]. For instance, in the 38 patients studied by Cohen-Gadol et al. [1] who underwent biopsy of spinal lesions, the overall duration of symptoms for patient with inflammatory processes was shorter than that for patients with neoplasms (mean 9.3 months versus 28.9 months, resp.).

In general, CSF profiles may be similar between spinal cord tumors and inflammatory processes [4]. More specifically, the presence of oligoclonal bands in CSF does not completely exclude the presence of spinal cord tumors with absolute certainty [5, 7]. On the other hand, oligoclonal bands in the CSF are typically absent in association with inflammatory processes other than MS [8, 9]. Moreover, in the aforementioned study by Cohen-Gadol et al. [1], other differences in CSF profiles did not reach any statistical significance between patients with intramedullary spinal cord tumors and those with inflammatory processes.

As the treatment of choice for inflammatory lesions of the spinal cord is nonsurgical management, accurate diagnosis of these lesions is paramount. This is of particular importance, because even small surgical biopsy procedures are not without complications. For instance, in 38 patients with intramedullary spinal cord lesions studied by Cohen-Gadol et al. [1], 21% of the patients experienced postoperative complications that ranged from CSF leaks and worsening neurological deficits to the need for reoperation. Thus, in many cases of questionable spinal cord lesions, empirical medical therapy may be a reasonable option, whereas surgical biopsy procedures may be reserved for cases refractory to medical therapy or for patients with progressive neurological deficits in spite of treatment. A trial of conservative treatment with high-dose steroids followed by repeat imaging studies may be therefore a prudent initial therapeutic algorithm for patients with mass-like lesions of the spinal cord.

In conclusion, idiopathic transverse myelitis may mimic spinal tumors by presenting as an intramedullary mass lesion of the spinal cord. The indications for spinal cord biopsy must take into account the clinical symptoms, the rate of progression of neurological deficits, and the imaging characteristics of the lesion. Surgical biopsy procedures carry certain postoperative risks of morbidity and may be reserved for cases with progressive neurological deficits refractory to empirical medical therapy.

## Figures and Tables

**Table 1:** Case reports of transverse myelitis presenting as intramedullary mass lesion.

Authors, yr	Sex, age (yr)	Symptoms	Level	Oligoclonal bands in CSF	Biopsy/surgery	Final diagnosis
Dhiwakar and Buxton, 2004 [3]	F, 36	Quadriparesis, paresthesia, urinary retention	C1–T2	Yes	Yes	MS

Brinar et al., 2006 [4]	M, 33	Weakness, paresthesia	C1–C3	Yes	No	MS
	F, 26	Pain, paresthesia, weakness, urinary retention	Medulla-C5	No	Yes	ADEM
	F, 33	Paresthesia, weakness	C3–C6	No	No	ADEM
	F, 36	Paresthesia, paraparesis, urinary incontinence	C1–C4	No	Yes	NMO
	M, 54	Pain, stiffness, paresthesia, paraparesis, urinary incontinence	T4–T6	Yes	No	ADEM

Habek et al., 2011 [5]	F, 43	Quadriparesis, urinary incontinence	Medulla-T1	Yes	Yes	NMO

Fanous et al. (current study)	M, 66	Pain, paresthesia	C2–C4	No	Yes	ITM

ADEM, acute disseminated encephalomyelitis; CSF, cerebrospinal fluid; F, female; ITM, idiopathic transverse myelitis; M, male; MS, multiple sclerosis; NMO, neuromyelitis optica; Yr, year(s).
